# New Hybrid Copper Nanoparticles/Conjugated Polyelectrolyte Composite with Antibacterial Activity

**DOI:** 10.3390/polym13030401

**Published:** 2021-01-27

**Authors:** Ignacio A. Jessop, Yasmín P. Pérez, Andrea Jachura, Hipólito Nuñez, Cesar Saldías, Mauricio Isaacs, Alain Tundidor-Camba, Claudio A. Terraza, Ingrid Araya-Durán, María B. Camarada, José J. Cárcamo-Vega

**Affiliations:** 1 Organic and Polymeric Materials Research Laboratory, Facultad de Ciencias, Universidad de Tarapacá. P.O. Box 7-D, Arica 1000007, Chile; yasmin.perezmorales@gmail.com (Y.P.P.); andreajachura17@gmail.com (A.J.); hipolitosergio@gmail.com (H.N.); 2 Facultad de Química y de Farmacia, Pontificia Universidad Católica de Chile, Santiago 7820436, Chile; casaldia@uc.cl (C.S.); misaacs@uc.cl (M.I.); 3 Research Laboratory for Organic Polymers (RLOP), Facultad de Química y de Farmacia, Pontificia Universidad Católica de Chile, Santiago 7820436, Chile; atundido@uc.cl (A.T.-C.); cterraza@uc.cl (C.A.T.); 4 Centro de Nanotecnología Aplicada, Facultad de Ciencias, Universidad Mayor, Santiago 8580745, Chile; ingrid.araya.duran@gmail.com; 5 Núcleo de Química y Bioquímica, Facultad de Estudios Interdisciplinarios, Universidad Mayor, Santiago 8580745, Chile; 6 Universidad de Tarapacá, P.O. Box 6-D, Arica 1000000, Chile; jjcarcamo@gmail.com

**Keywords:** conjugated polyelectrolyte, copper nanoparticles, nanoparticles stabilization, nanoparticles-polyelectrolyte hybrid composite, antibacterial activity

## Abstract

In the search for new materials to fight against antibiotic-resistant bacteria, a hybrid composite from metallic copper nanoparticles (CuNPs) and a novel cationic π-conjugated polyelectrolyte (CPE) were designed, synthesized, and characterized. The CuNPs were prepared by chemical reduction in the presence of CPE, which acts as a stabilizing agent. Spectroscopic analysis and electron microscopy showed the distinctive band of the metallic CuNP surface plasmon and their random distribution on the CPE laminar surface, respectively. Theoretical calculations on CuNP/CPE deposits suggest that the interaction between both materials occurs through polyelectrolyte side chains, with a small contribution of its backbone electron density. The CuNP/CPE composite showed antibacterial activity against Gram-positive (*Staphylococcus aureus* and *Enterococcus faecalis*) and Gram-negative (*Escherichia coli* and *Salmonella enteritidis*) bacteria, mainly attributed to the CuNPs’ effect and, to a lesser extent, to the cationic CPE.

## 1. Introduction

Humanity is often faced with biosecurity threats, such as malaria, tuberculosis, smallpox, etc., which have caused millions of deaths in the past. Recently, the SARS-CoV-2 virus strain that causes the coronavirus disease (COVID-19) has been strongly impacting the global society, economy, and public health since 2019 to date [1,2,3]. New antibiotics and vaccines are continuously being tested to fight against old and emerging diseases caused by infectious agents, such as viruses, parasites, fungi, and bacteria, due to their increasing global proliferation. During the last decades, a significant public health issue has been diseases promoted by drug-resistant bacterial strains, which have emerged due to overuse and misuse of antibiotics and disinfectants [4,5,6,7]. Therefore, there is an urgent need to develop new antibacterial agents to treat infections caused by resistant strains [8,9].

An interesting alternative that has shown a broad spectrum of biocidal activities is metal nanoparticles (NPs) [10,11,12]. The antimicrobial mechanism of NPs is related to the disruption of the bacterial outer membrane, the generation of reactive oxygen species (ROS), the penetration of the cell membrane, and the occurrence of a series of intracellular processes, which include interactions with proteins and DNA [4,5,10]. Among the metal NPs that have been tested as antibacterial agents, the copper nanoparticles (CuNPs) stand out because they are cost-efficient alternatives to other noble-metal NPs such as silver NPs (AgNPs) and gold NPs (AuNPs); however, CuNPs are very reactive and are easily oxidized to form copper oxides when exposed to air [11,13,14]. Protecting or capping agents such as surfactants or polymers have been used to prevent the oxidation and aggregations of CuNPs, leading to stabilized CuNP/protecting agent composites used in a variety of technological applications [15,16,17,18].

Conjugated polyelectrolytes (CPEs) are another class of materials that have received great attention during the last decade due to their light-harvesting capacity, efficient energy transfer, and singlet oxygen generation properties [19,20,21], making them suitable for biomedical applications, such as fluorescence imaging, biosensing, and biocidal and photodynamic therapy [22,23,24,25,26,27]. The delocalized electronic backbone and pendant ionic groups of CPEs determine their optical properties and allow them to interact with the biological targets, respectively. The antimicrobial activity of CPEs is related to the disruption of the germ membrane structure and ROS promotion under light irradiation [28,29]. The incorporation of cationic side groups into CPEs has been shown to play an essential role in killing both Gram-positive and Gram-negative bacteria, as they interact with their negatively charged envelopes [30,31,32].

The production of hybrid NPs/polymers or CPE composites with combined properties has been studied in several studies [17,33,34,35,36,37,38,39]. However, there is scarce literature on the synthesis and application of CuNP/CPE composites [40]. In this sense, the aim of this study is to develop a new conjugated polyelectrolyte, poly((9,9′-bis(6′’-(*N*,*N*,*N*-trimethylammonium)hexyl)-9*H*-fluorene-2,7-diyl)-alt-(2-(6-(*N*,*N*,*N*-trimethylammonium)hexyl)-2*H*-naphtho[2,3-d][1–3]triazole-4,9-diyl) tribromide) (CPE), and to explore its properties as a stabilizing agent of CuNPs. The CPE structure was designed to produce a material (1) for which the absorption maximum does not overlap with the CuNP plasmon resonance band; (2) with flexible and cationic side chains attached to both monomers to promote the solubility of CPE in water and electrostatic interactions with the CuNPs; and (3) with a partially twisted structure (see Section 3.2.) to minimize the inter-chain aggregation [41,42], which would also contribute to its solubility in an aqueous medium.

The CuNPs were prepared in an aqueous medium by chemical reduction of copper ions with hydrazine hydrate in a CPE aqueous solution to give a stable CuNP/CPE suspension. The composite was characterized by UV-vis and fluorescence spectroscopy, SEM/FESEM techniques, and theoretical calculations. The antibacterial activity of the new CuNP/CPE against Gram-positive (*Staphylococcus aureus* and *Enterococcus faecalis*) and Gram-negative (*Escherichia coli* and *Salmonella enteritidis*) bacteria was tested via a dilution method, showing its potential for use in biomedical applications focused on the development of medical supplies such disposable masks, non-woven fabrics, patches, etc.

## 2. Materials and Methods

### 2.1. Materials

All reagents and solvents were used without further purification. 1,6-Dibromohexane, tetrakis(triphenylphosphine)palladium (0) (Pd[PPh_3_]_4_), tetrabutylammonium bromide (TBAB), phenylboronic acid, bromobenzene, and polyvinylpyrrolidone (average mol wt 10,000) (PVP10) were purchased from Sigma-Aldrich (Milwaukee, WI, USA). 2,3-Diaminonaphthalene, 2,7-dibromofluorene, 1,1′-bis(diphenylphosphino)ferrocene-palladium(II) dichloride dichloromethane complex (Pd[dppf]Cl_2_·CH_2_Cl_2_), bis(pinacolato)diboron (B2pin2), and potassium acetate (KOAc) were obtained from AK Scientific, Inc. (San Francisco, CA, USA). Bromine (Br_2_), sodium nitrite (NaNO_2_), acetic acid (HAc), triethylamine (NEt_3_), potassium hydroxide (KOH), potassium carbonate (K_2_CO_3_), ammonium hydroxide solution (NH_4_OH), trimethylamine (40% solution in water) (NMe_3_), copper(II) chloride dihydrate (CuCl_2_·2H_2_O), potassium iodide (KI), hydrazine hydrate (80% in water) (N_2_H_4_·H_2_O), and all solvents were acquired from Merck (Darmstadt, Germany). Cetyltrimethylammonium bromide (CTAB) was purchased from BiosLabChile (Santiago, Chile). Monomers 4,9-dibromo-2-(6-bromohexyl)-2*H*-naphtho[2,3-d][1–3]triazole (M1) and 2,2′-(9,9-bis(6-bromohexyl)-9*H*-fluorene-2,7-diyl)bis(4,4,5,5-tetramethyl-1,3,2-dioxaborolane) (M2) were prepared according to previously described procedures [43,44].

*Staphylococcus aureus* ATCC25923 (*S. aureus*), *Enterococcus faecalis* ATCC29212 (*E. faecalis*), *Escherichia coli* ATCC25922 (*E. coli*), and *Salmonella enteritidis* ATCC13076 (*S. enteritidis*) were kindly provided by H. Nuñez, Universidad de Tarapacá, Arica, Chile. Tryptic soy broth (TSB) medium was used in the cell culture.

### 2.2. Measurements

^1^H and ^13^C NMR spectra were recorded on a Bruker AVANCE III HD 400 MHz spectrometer (Bruker Corporation, Karlsruhe, Germany) in deuterated solvents. Chemical shifts were reported as δ values (ppm) relative to an internal tetramethylsilane (TMS) standard. Number-average (*Mn*) and weight-average (*Mw*) molecular weights were determined by size exclusion chromatography (SEC) on a Wyatt Technology Dawn EOS HPLC (Wyatt Technology, Santa Barbara, CA, USA) instrument equipped with a high-pressure liquid chromatography (HPLC) Knauer pump, three PLgel 5 μMixed-C columns, and static light-scattering (EA-02 Dawn Eos Enhanced Optical System) detector. The flow rate was 1.0 mL·min^−1^ using tetrahydrofuran (THF) as an eluent at 25 °C. The samples were prepared at 1.0 mg·mL^−1^ in THF and were filtered through a 0.45 μm nylon filter. The calibration curve was made with a series of monodisperse polystyrene standards. UV-vis absorption spectra were obtained using a Shimadzu UV−1800 spectrophotometer (Shimadzu Corporation, Kyoto, Japan) using quartz cells of 1 cm path length. Fluorescence spectra were recorded from a LS55 PerkinElmer fluorescence spectrometer (PerkinElmer, Waltham, MA, USA) using a quartz cuvette cell of 1 cm path length fluorescence. The samples were excited at the wavelength of maximum absorption of the polymers in solution. The images were recorded from a scanning electron microscope (SEM) fitted with a Jeol JCM-6000 energy dispersive X-ray (EDX) detection system (Jeol Co., Akishima, Tokyo, Japan), operating at an acceleration voltage of 15 kV. The FESEM images were obtained from a Quanta FEG 250 ESEM equipped with a DF STEM detector microscope (FEI, Czech Republic) operating at an acceleration voltage of 20 kV. The images were obtained from CuNP/CPE deposits. The corresponding suspensions were filtered through 0.45 μm nylon filters, and the filtered solids were rapidly stored under a nitrogen atmosphere before use. The filtered deposits were resuspended in DMSO, and a sample of the dispersion was dripped onto a copper grid coated with a Formvar and a carbon film.

### 2.3. Synthesis of Conjugated Polymer (CP) and Polyelectrolyte (CPE)

Synthesis of poly[(9,9′-bis(6′’-bromohexyl)-9H-fluorene-2,7-diyl)-alt-(2-(6-bromohexyl)-2H-naphtho[2,3-d][1–3]triazole-4,9-diyl)] (CP): a mixture of M1 (0.35 mmol), M2 (0.35 mmol), and Pd[PPh3]4 (6·10^−3^ mmol) was purged under a steady stream of N_2_ for 30 min at room temperature. Degassed toluene (7.0 mL) and K_2_CO_3_ (2 M, 4.5 mL) were added, and the reaction mixture was stirred and heated at 110 °C for 48 h under N_2_ atmosphere. An end-capping procedure was performed using phenylboronic acid and bromobenzene. After cooling to room temperature, the reaction mixture was poured into methanol/acidified water (9:1 *v*/*v*), and the resulting solid was filtered through a Soxhlet thimble. The solid was washed using a Soxhlet apparatus with acetone and n-hexane. The polymer was extracted with chloroform, and the solution was concentrated to 5–10 mL and poured into methanol. The precipitated was filtered through a 0.45 μm nylon filter and vacuum-dried to give a red solid (yield: 49%). ^1^H NMR (400 MHz, CDCl_3_) δ 8.24–7.24 (m, 10H), 4.94–4.67 (m, 2H), 3.43–3.08 (m, 6H), 2.23–2.11 (m, 2H), 1.88–0.92 (m, 26H).

Synthesis of poly[(9,9′-bis(6″-(N,N,N-trimethylammonium)hexyl)-9H-fluorene-2,7-diyl)-alt-(2-(6-(N,N,N-trimethylammonium)hexyl)-2H-naphtho[2,3-d][1–3]triazole-4,9-diyl) tribromide] (CPE): the precursor CP was dissolved in 5 mL of THF/MeOH (4:1 *v*/*v*), and then 3.0 mL of NMe3 (40 wt % solution in water)/MeOH (1:1 *v*/*v*) was added dropwise with stirring. The reaction mixture was stirred for 24 h at room temperature and then was poured into diethyl ether. The precipitated was filtered through a 0.45 μm nylon filter and vacuum-dried to give an orange solid (yield: 69%). ^1^H NMR (400 MHz, DMSO-d_6_) δ 8.33–7.35 (m, 10H), 5.04–4.71 (m, 2H), 3.34 (s, 27H), 3.11–2.92 (m, 6H), 2.32–0.96 (m, 28H).

### 2.4. Preparation of CuNP/CPE Composite

An aqueous CPE solution was prepared by dissolving the CPE (5.0, 15, or 30 mg) in 1.0 mL of DMSO. Distilled water was slowly added to the mixture at 50 °C in an ultrasonic bath until the solution’s total volume was 25.0 mL. The aqueous CPE solution was used as a solvent to prepare 10.0 mL of a 5 mM solution of CuCl_2_·2H_2_O and 10.0 mL of an 80 mM solution of N_2_H_4_·H_2_O. Equal volumes of copper salt and hydrazine solutions were mixed into a 100 mL round-bottom capped flask. The pH of the resulting mixture was adjusted to 10 using ammonia solution, and the reaction was allowed to proceed at room temperature without stirring and without adding inert gases, given the N_2_ generation as a by-product. After about 4 h, a reddish-brown CuNP/CPE suspension was observed, which can be stored for several weeks for future uses. For comparison, CuNP/CTAB and CuNP/CTAB/PVP systems were synthesized according to Wu et al. and Pham et al.’s reported procedures, respectively [15,16]. The copper salt and reducing agent concentrations were equal in all experiments.

### 2.5. Computational Details

The geometry of the CPE repeating unit was fully optimized at the density functional theory (DFT) level using the Gaussian16 computational package [45]. Becke’s three parameters, nonlocal hybrid exchange potential with the nonlocal correlation functional of Lee, Yang, and Parr (B3LYP) [46,47,48] was implemented without any symmetry restriction, with a triple-ζ basis set TZVP [49] for all atoms. Each dihedral angle of the aromatic rings was scanned by internal rotations to explore the potential energy surface and to find the lowest energy conformation. The trimer conformation was then optimized at the same level of theory. To study the trimer and copper nanoparticles interaction, a cluster of eight copper atoms was located at different trimer sites to identify the most stable conformation. No symmetry restrictions were applied, while the copper atoms were described with a relativistic effective core potential basis set with pseudopotentials (LANL2DZ [50]). A tight SCF convergence criterion (10−8 a.u.) was used in all calculations. The charge distribution of intermolecular interactions was calculated by the natural population analysis (NPA) method [51], as implemented in Gaussian16. The interaction energy (E_int_) was defined according to the following expression: E_int_ = ET-Cu_8_ − ET (T-Cu_8_) − ECu_8_ (T-Cu_8_), which represents the energy difference between the complex and the energies of constituent monomers, the trimer (T), and the copper cluster. It is well known that the estimation of E_int_ with finite basis sets introduces an error due to the basis set superposition (BSSE). The BSSE is related to using different numbers of basis functions to describe the complex and monomers for the same basis set. BSSE-corrected interaction energies were computed using Boys–Bernardi counterpoise correction scheme [52].

### 2.6. Preparation of Microorganism–CuNP/CPE Suspensions

Microbiological assays were carried out with the serial dilution method by evaluating the visible growth (or turbidity) of bacteria in TSB culture medium. Briefly, 9.0 mL of TSB were dispensed into ten autoclaved test tubes (labeled A–J) and left for 24 h at 37 °C in a stove. Then, 1.0 mL of CPE or CuNP/CPE aqueous solution/suspension (1.0 mg·mL^−1^) was added and thoroughly mixed with the first dilution tube. From this first tube, 1.0 mL was transferred to the second dilution tube and so on until the 9th tube. Tube 10 was used as a control (without CPE or CuNP/CPE). One milliliter of bacterial strain (10^9^ CFU·mL^−1^, samples 1–4) was added to each tube, which was sterilized by flaming its neck, cotton wool-plugged, and incubated at 37 °C in the dark or under a white LED light (20 mW·cm^−2^) for 24 h. The effect of DMSO concentration (used to dissolve CP) on the bacterial culture growth was also assessed by using 1.0 mL of DSMO aqueous solution (4% *v*/*v*) instead of the bacterial growth inhibitor solution. Turbidity was analyzed by visual inspection of the tubes. All experiments were performed in duplicate.

## 3. Results and Discussion

### 3.1. Synthesis and Characterization of CuNP/CPE Composite

**Scheme 1:**
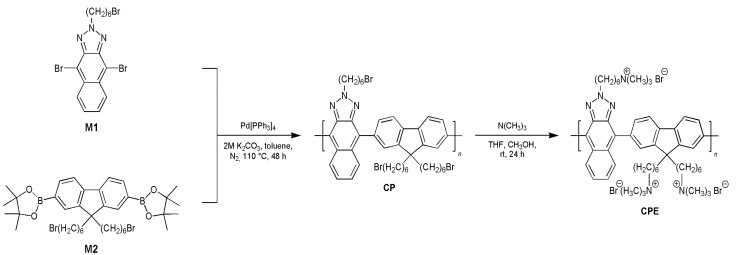
Synthetic route for conjugated polymer (CP) and conjugated polyelectrolyte (CPE).

The synthesis of the new CPE is depicted in Scheme 1. First, the nonionic π-conjugated polymer CP was prepared via Suzuki polycondensation reaction from the monomers M1 (an electron-acceptor naphthotriazole derivative) and M2 (an electron-donor fluorene derivative), obtaining a red solid in 49% yield. Size-exclusion chromatography (SEC) analysis showed that *Mn*, *Mw*, and polydispersity index (PDI) of CP were 16,800 g·mol^−1^, 14,300 g·mol^−1^, and 1.2, respectively. CP was then post-functionalized with trimethylamine (quaternization reaction) according to a previous procedure [53] to obtain the π-conjugated polyelectrolyte CPE as an orange solid in 69% yield. CP and CPE were characterized by ^1^H NMR spectroscopy, showing a good agreement with the expected structures (Figures S1 and S2).

The CuNP/CPE composite was obtained by modifying the method reported by Pham et al. [15], where the reduction of cupric chloride with hydrazine occurs in the presence of CTAB and polyvinylpyrrolidone (PVP), acting as a stabilizing agent and preventing the aggregation of the CuNP–CTAB system. Since the CPE was poorly soluble in an aqueous medium, it was first dissolved in 1.0 mL of DMSO, and then, the mixture was diluted with distilled water to a total volume of 25 mL at 50 °C and under sonication. The low solubility of CPE in water reflects its high tendency to aggregate in this medium [41,42] despite the proposed structural design. The aqueous CPE solution was used as a solvent of the copper salt and hydrazine solutions. Both solutions were mixed, and the pH was adjusted to 10 to give the CuNP/CPE composite after 4 h without stirring. An ammonia solution was used to set the solution pH 10 to avoid the formation of cuprous or cupric oxides [15,16]. The CuNP/CPE composite was synthesized using different amounts of CPE: 5.0 mg, 15 mg, and 30 mg (Figure S3a–c in the Supplementary Materials). A stable CuNP/CPE suspension was observed when 5.0 mg of CPE was used, while CPE-stabilized CuNPs tend to precipitate with higher amounts of polyelectrolyte because of its self-aggregation.

As stated by Pham et al. [15], the nitrogen gas by-product creates an inert atmosphere which protects CuNPs against oxidation during the reaction. With this protection, the capped CuNP/CPE suspensions were stable after several weeks. To evaluate their oxidation resistance, CuNP/CPE suspensions were left uncapped at room temperature. After 24 h, the control suspension (a capped suspension) remained unchanged while the suspended (5.0 mg of CPE) or aggregated (15 mg and 30 mg of CPE) composites in the uncapped flasks dissolved and turned into green solutions (Figure S3d), indicating CuNP oxidation in the aqueous medium.

According to the proposed mechanism of CuNP stabilization with capping agents [15,16,54,55,56,57,58], the ionic groups in the alkyl side chains of CPE would be mainly responsible for CuNP stabilization; however, the high electron density on the surface of the π-conjugated polyelectrolyte could also play a role in the CuNP stabilization. To test this hypothesis, theoretical calculations were performed, and the results are detailed in Section 3.2.

**Figure 1:**
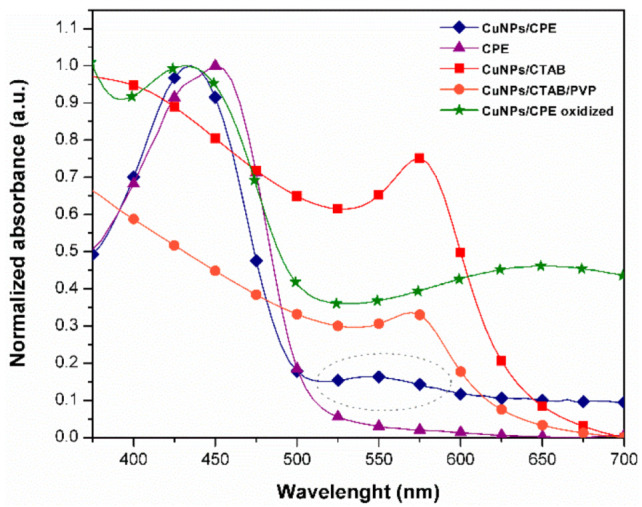
UV-vis spectra of the CPE and the copper nanoparticles (CuNP)/CPE (stabilized and oxidized), CuNP/cetyltrimethylammonium bromide (CTAB), and CuNP/polyvinylpyrrolidone (PVP)/CTAB composites in aqueous suspensions (10^−4^ mol·L^−1^).

UV-vis spectra of the CPE and CuNP/CPE systems along with the spectra of CuNPs prepared in the presence of CTAB and CTAB–PVP for comparison are shown in Figure 1. As seen, an absorption band in the range of 500–600 nm is observed in all spectra except that of pure CPE, which corresponds to the surface plasmon resonance produced by the metallic CuNPs [14,15,16,59]. The composite plasmonic band is broader compared to the bands of the CuNP/CTAB and CuNP/CTAB/PVP systems, which would imply a larger CuNP size distribution and a lower CPE stabilizing capability. In contrast to the comparison systems, which presented more defined CuNP bands, the lower absorption of CuNPs in the CuNP/CPE spectrum could be attributed to the high absorption of CPE that hides the peaks of the copper nanoparticles. A signal at 450 nm is observed in CPE spectra, while in CuNP/CPE, the spectra are centered at 435 nm. This band originates from the intramolecular charge transfer (ICT) process between the electron-donor (fluorene) and electron-acceptor (naphthotriazole) units along with the CPE structure [60,61,62]. The spectrum of an oxidized CuNP/CPE suspension was also recorded, showing the two absorption bands expected but shifted in relation to the maximum peaks of the stabilized suspension. The first band at 432 nm is due to CPE absorption, while the second band at 651 nm is red-shifted in relation to the comparison systems. This band shifting could be related to the corrosion and dissolution processes of the NPs in the presence of oxygen [33]. In this process, copper ions are released from the polymer matrix to the solution, which could interact with dissolved oxygen, with bromide ions, or with the ammonium ions of the reaction medium or in the CPE side groups to generate cuprous or cupric oxides, copper (I or II) bromides, or even copper ammonia-like complexes, respectively [18,63,64,65].

**Figure 2:**
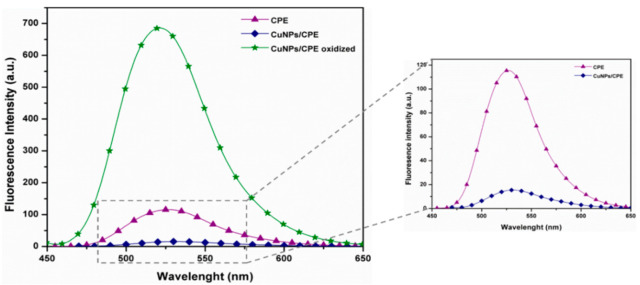
Fluorescence spectra of CPE and CuNP/CPE (stabilized and oxidized) composites in aqueous suspensions (10^−6^ mol·L^−1^).

The fluorescence spectra of CPE and CuNP/CPE systems (stabilized and oxidized) were also recorded (Figure 2). As seen, the CPE fluorescence was quenched by CuNPs, which reflects their effective interaction [38]. When the CuNP/CPE suspension was left to oxidize in the air, the fluorescence intensity tended to increase even more than the CPE fluorescence intensity, which could be attributed to the copper ammonia-like complex emission or the CuO NP formation, for which an emission at about 520 nm has been reported in previous works [66,67].

**Figure 3:**
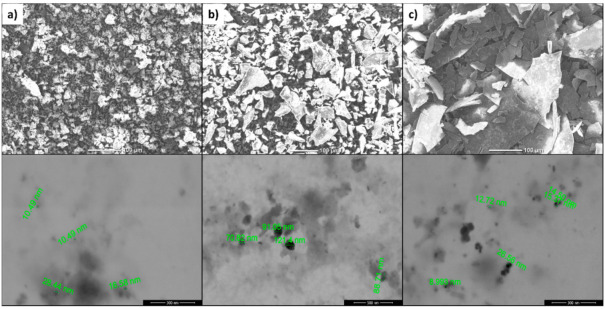
SEM (**up**) and FESEM (**down**) images of CuNP/CPE composites with different amounts of CPE: (**a**) 5 mg, (**b**) 15 mg, and (**c**) 30 mg.

The CuNP/CPE samples with different CPE loads were characterized by SEM and FESEM techniques (Figure 3a–c). The SEM images showed that, as the amount of CPE increases, the polyelectrolyte aggregates into sheet structures, which would explain the observed decrease in water solubility of CPE and CuNP/CPE composites. The FESEM images exhibited copper nanodomains of relatively spherical shapes being embedded in the CPE matrix, which tended to form clusters as the concentration of CPE increased. Clustering may also be related to the way that the measurement was conducted (Section 2.2). As demonstrated by Pham et al. [15], CuNPs can form large aggregates in CTAB. To avoid such aggregation, the authors added PVP to the reaction medium to prevent CuNPs covered by CTAB bilayers from forming large clusters that could lead to partial oxidation of the nanoparticles. As seen in FESEM images, the CuNPs were randomly distributed in the CPE, acting as both CTAB and PVP in a single material as expected.

### 3.2. Theoretical Calculations

**Figure 4:**
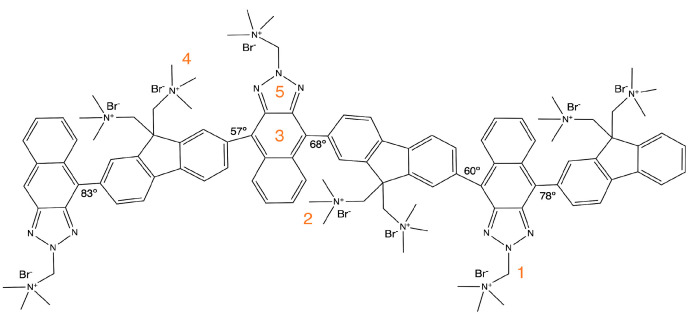
Dihedral angles of the optimized CPE trimer and potential binding sites with CuNPs.

To gain insight into the interactions between CuNPs and CPE in solid state, computational calculations were carried out. Firstly, the monomer ground state geometry was optimized by performing the internal rotation of dihedral angles linking the two units at the B3LYP/TZVP level of theory. Shorter tetramethylammonium bromide groups replaced the hexyltrimethylammonium bromide chains. Subsequently, the trimer structure (selected as a representative of the polymer) was optimized at the same level. The vibrational analysis confirmed the optimized geometries as local minima. As expected, the trimer-optimized geometry presents a twisted conformation because of the dihedral angles (close to 60°) between the monomers (Figure 4), which directly impacts the polymer stability and the delocalization of the electronic density. Figure S4 shows a plot of the trimer frontier orbitals, mainly centered on the benzotriazole moieties.

The potential coordination sites where the metal ion could attach and generate complexes were considered for the binding analysis. The Cu_8_ cluster was placed next to the electron-rich regions in the trimer structure (1–5 sites in Figure 4) to build the starting geometries of T-Cu_8_ complexes for optimization. To search for alternative local minima, three initial conformations were tested at each coordination site. Table 1 lists the most stable E_int_ at the selected coordination sites, the distances between the Cu_8_ cluster and the anchor atom, and the charge of optimized complexes.

**Figure 5:**
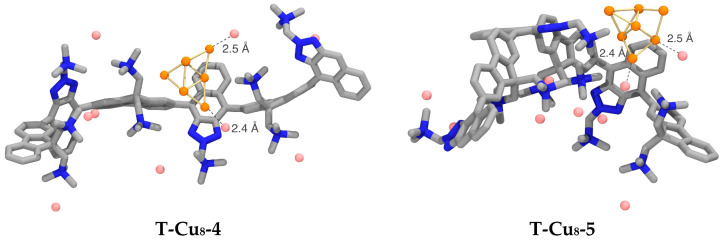
The optimized structures of complex T-Cu_8_-4 and T-Cu_8_-5.

As seen in Table 1 and Figure 5, the E_int_ analysis shows that the most stable T-Cu_8_ complexes are formed when the copper cluster interacts with sites 4 (T-Cu_8_-4) and 5 (T-Cu_8_-5) of the trimer, with a difference of approximately 3.5 kcal·mol^−1^ in favor of site 4. T-Cu_8_-4 and T-Cu_8_-5 exhibited double stabilization interactions with the negative counter ions electrostatically attached to the quaternary ammonium side groups, which is consistent with the literature [15,16].

Regarding the frontier orbitals, the HOMO is localized at the copper cluster while the LUMO concentrates at the benzotriazole (Figure S5). The total NPA charge analysis of the copper cluster (Δq_cluster_) indicated that the trimer and Br^−^ ions transferred part of their charge to the cluster. Complex T-Cu_8_-5 presented the greatest amount of transferred electron density, with approximately 90% more transferred charge than complex T-Cu_8_-3, the less stable of the series, associated with stronger interaction and stability. This implies that the CPE electron-rich backbone also contributes to the CuNP stabilization.

**Figure 6:**
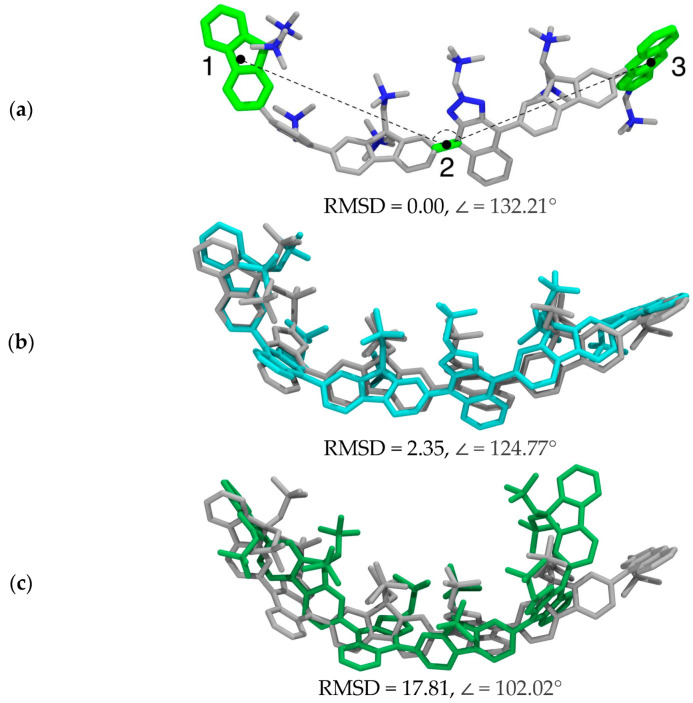
(**a**) The bending angle (∠) calculated as the angle between the vectors of the external fluorene mass centers and RMSD of the trimer in (**b**) T-Cu8-4 and (**c**) T-Cu8-5: copper and bromine atoms were excluded from the analysis. The optimized structure of the trimer is colored in gray.

One interesting finding is that, although the trimers in T-Cu_8_-4 and T-Cu_8_-5 have similar E_int_, the trimer in T-Cu_8_-4 tends to adopt a more extended conformation. The trimer structure in both complexes was compared to the isolated optimized trimer by the root mean square deviation (RMSD) and internal bending angle. H, Br, and Cu atoms were excluded from this analysis. The bending angle (∠) was calculated as the angle between the vectors of the external mass centers with an initial value of 132° (Figure 6). The trimer in T-Cu_8_-4 bent at 7° and the RMSD were very close to the isolated trimer structure, while the trimer in T-Cu_8_-5 bent at a higher degree (30°) and the RMSD increased to 17.81. This results indicate that CPE tends to adopt different conformations to stabilize the CuNPs, mainly through proper orientation of the side chain’s negative counter ions and, secondly, of the electron-acceptor monomer. It should be noted that the calculations and simulations herein represent the interactions between the components of the composite in a deposit, with potential biotechnological applications such as medical supplies.

### 3.3. Microbiological Assays

The antibacterial activity of CuNP/CPE hybrid composite against two Gram-positive (*S. aureus* and *E. faecalis*) and two Gram-negative bacteria (*E. coli* and *S. enteritidis*) was studied using a broth dilution method. Firstly, the bacteria cultures were incubated for 24 h under aerobic conditions in the presence of an aqueous DMSO solution of the same concentration as used to prepare the CPE or CuNP/CPE suspensions. Turbidity was observed in all test tubes, demonstrating that DMSO does not contribute to the samples’ bacterial growth inhibition effect.

Secondly, the antibacterial activity of CPE solution was analyzed after 24 h of incubation in the dark, observing bacterial growth throughout the concentration range (Table 2 and Figure S6). It should be noted that a significant amount of sediment was observed in the tubes at the concentration of 1·10^−1^ mg·mL^−1^ for all bacterial strains. Apparently, the CPE does not possess an inherent bacterial growth inhibition capacity. However, it has the potential to establish electrostatic and hydrophobic interactions through its quaternary ammonium side groups with the outer envelopes of Gram-positive and Gram-negative bacteria given their negative surface charge, which affects the integrity of bacterial cells and which also promotes their aggregation and precipitation [28,32,68,69]. The bacterial strains were also incubated with CPE under white light irradiation (Figure S7). Less turbidity was visualized in all test tubes, showing that CPE has a certain antibacterial activity related to ROS generation after activation by light [7,35,70].

Finally, the bacterial growth inhibition effect of CPE/CuNPs was analyzed (Table 1 and Figure S8). No turbidity was observed in all bacterial strains samples at the concentration of 1 × 10^−1^ mg·mL^−1^ after incubation in the dark. This fact indicates that the composite antimicrobial activity is mostly attributable to the CuNPs. According to the literature, the copper ions generated by the corrosion process of CuNPs are released into the medium, which interferes with the integrity of the outer membrane and diffuses into the bacteria, causing massive oxidative stress that leads to a functionality disruption [10,33,70]. Growth inhibition was observed in Gram-negative bacteria at a lower concentration (1 × 10^−2^ mg·mL^−1^) compared to Gram-positive bacteria. This is consistent with previous reports that showed that Gram-positive bacteria are more resilient to the biocidal effect of NPs, including CuNP/CTAB systems [10,71]. As stated before, the interactions between CPE and the outer envelopes of bacteria also play a role in CuNP/CPE composite antibacterial activity. This is reasonable since tests under irradiation showed that turbidity decreased in all tubes, reflecting the combined action of CuNPs and CPE. According to the obtained results, the CuNP/CPE composite could potentially become an alternative for the fight against bacterial infections.

## 4. Conclusions

A hybrid composite based on metallic copper nanoparticles stabilized by a conjugated donor-acceptor polyelectrolyte (CuNP/CPE) with antibacterial activity was synthesized and characterized. UV-vis and fluorescence studies demonstrate the effective formation of this new CuNP/CPE hybrid material. Theoretical calculations indicate that CuNP stabilization in solid state occurs mainly through the negative counter ions of the lateral quaternary ammonium groups and, to a lesser extent, by the electron-density at the CPE surface concentrated in the electron-acceptor naphthotriazole monomer. The SEM micrographs show that CPE adopts a rigid laminar structure, which explains its partial solubility in an aqueous medium. Additionally, the FESEM micrographs indicate a random distribution of the nanoparticles on the CPE surface. The antibacterial activity of both CPEs and CuNP/CPE composites was tested in vitro against two Gram-positive bacterial strains (*E. faecalis* and *S. aureus*) and two Gram-negative bacteria (*S. enteritidis* and *E. coli*) in the presence and absence of white light. The CPE exhibited no bactericidal activity, although a slight effect was visualized under light irradiation. The CuNP/CPE system showed antimicrobial capacity against the four strains at 1·× 10^−1^ mg·mL^−1^ and against the Gram-negative bacteria at 1·× 10^−2^ mg·mL^−1^ when irradiated with white light. The antibacterial effect of the CuNP/CPE composite is mainly attributed to the CuNPs and, secondly, due to the CPE. The results suggest that this new hybrid material could be used in the development of medical supplies for biomedical applications.

## Figures and Tables

**Table 1:** Theoretical parameters calculated to establish the potential coordination sites between CuNPs and CPE.

Complex	Anchor Bond	d_X–Cu_^a^	q_x_^b^	q_Cu_^b^	Δq_cluster_^b^	−E_int_^c^
1	Cu–N	2.074	−0.353	0.305	−0.234	17.231 (29.958)
2	Cu–Br	2.407	−0.727	0.376	−0.399	37.278 (49.935)
3	Cu–C	2.394	−0.257	0.317	−0.056	18.303 (24.716)
4	Cu–Br	2.456	−0.744	0.33	−0.572	45.984 (60.315)
5	Cu–Br	2.449	−0.726	0.322		
	Cu–Br	2.417	−0.717	0.306	−0.624	42.495 (56.552)
	Cu–Br	2.455	−0.651	−0.012		

^a^ Bond distances in Å. ^b^ Natural population analysis (NPA)-derived atomic charges of the anchor atom q_x_, the bonded gold atom q_Cu,_ and the total charge of the metal cluster Δq_cluster_ in a.u. ^c^ Interaction Energy (E_int_, kcal·mol^−1^) for the studied complexes with basis set superposition (BSSE): corrected and uncorrected (in parenthesis).

**Table 2:** Turbidity for different concentrations of CPE or CuNP/CPE after 24 h in the dark.

Bacterial Strain	CPE	CuNP/CPE	CPE	CuNP/CPE	CPE	CuNP/CPE
	1 × 10^−1^ mg·mL^−1^		1 × 10^−2^ mg·mL^−1^		1 × 10^−3^ mg·mL^−1^	
*E. coli*	+	-	+	-	+	+
*S. enteriditis*	+	-	+	-	+	+
*S. aureus*	+	-	+	+	+	+
*E. faecalis*	+	-	+	+	+	+

Positive (+) = turbidity (growth); negative (-) = no turbidity (no growth).

## Data Availability

The data presented in this study are available on request from the corresponding author.

## Supplementary Materials

The following are available online at https://www.mdpi.com/2073-4360/13/3/401/s1, Figure S1. ^1^H NMR spectra of CP entry in CDCl_3_; Figure S2. ^1^H NMR spectra of CPE entry in DMSO-*d_6_*; Figure S3. Images of the reaction flasks containing the CuNPs and different amounts of CPE: (a) 5 mg, (b) 15 mg, and (c) 30 mg. (d) Suspension after 48 h uncapped; Figure S4. (a) HOMO and (b) LUMO frontier orbitals of CPE at the B3LYP/TZVP level; Figure S5. (a) HOMO and (b) LUMO frontier orbitals of complexes T-Cu_8_-4 and T-Cu_8_-5; Figure S6. Images of the *S. enteriditis* bacteria cultures incubated with the CPE composite for 24 h (a) in the dark and (b) under irradiation; Figure S7. Image of an experiment run under white light irradiation; Figure S8. Images of the *S. aureous* bacteria cultures incubated with the CuNP/CPE composite for 24 h (a) in the dark and (b) under irradiation.
