# Supplementary material for: New Hybrid Copper Nanoparticles/Conjugated Polyelectrolyte Composite with Antibacterial Activity
Source: Polymers (Basel). 2021 Jan 27;13(3):401. doi: 10.3390/polym13030401 (PMC7865910; doi:10.3390/polym13030401)
Supplement: Supplementary file 1 [file polymers-13-00401-s001.pdf]

polymers

Electronic Supplementary Information - ESI

New Hybrid Copper Nanoparticles/Conjugated Polyelectrolyte Composite with Antibacterial Activity

Ignacio A. Jessop [1,*], Yasmín P. Pérez [1], Andrea Jachura [1], Hipólito Nuñez [1], Cesar Saldías [2], Mauricio Isaacs [2], Alain Tundidor-Camba [3], Claudio A. Terraza [3], Ingrid Araya-Durán [4], María B. Camarada [4,5], José J. Cárcamo-Vega [6]

[1] Organic and Polymeric Materials Research Laboratory, Facultad de Ciencias, Universidad de Tarapacá. P.O. Box 7-D, Arica 1000007, Chile; iajessop@uta.cl (I.A.J), yasmin.perezmorales@gmail.com (Y.P.P), andreajachura17@gmail.com (A.J.), hipolitosergio@gmail.com (H.N)
[2] Facultad de Química y de Farmacia, Pontificia Universidad Católica de Chile, Santiago 7820436, Chile; casaldia@uc.cl (C.S), misaacs@uc.cl (M.I)
[3] Research Laboratory for Organic Polymers (RLOP), Facultad de Química y de Farmacia, Pontificia Universidad Católica de Chile, Santiago 7820436, Chile.; atundido@uc.cl (A.T.-C.); cterraza@uc.cl (C.A.T.)
[4] Centro de Nanotecnología Aplicada, Facultad de Ciencias, Universidad Mayor, Santiago 8580745, Chile; ingrid.araya.duran@gmail.com (I.A.-D.), maria.camarada@umayor.cl (M.B.C)
[5] Núcleo de Química y Bioquímica, Facultad de Estudios Interdisciplinarios, Universidad Mayor, Santiago 8580745, Chile.
[6] Universidad de Tarapacá, P.O. Box 6-D, Arica 1000000, Chile; jjcarcamo@gmail.com (J.J.C.-V.)
* Correspondence: iajessop@uta.cl (I.A.J.)

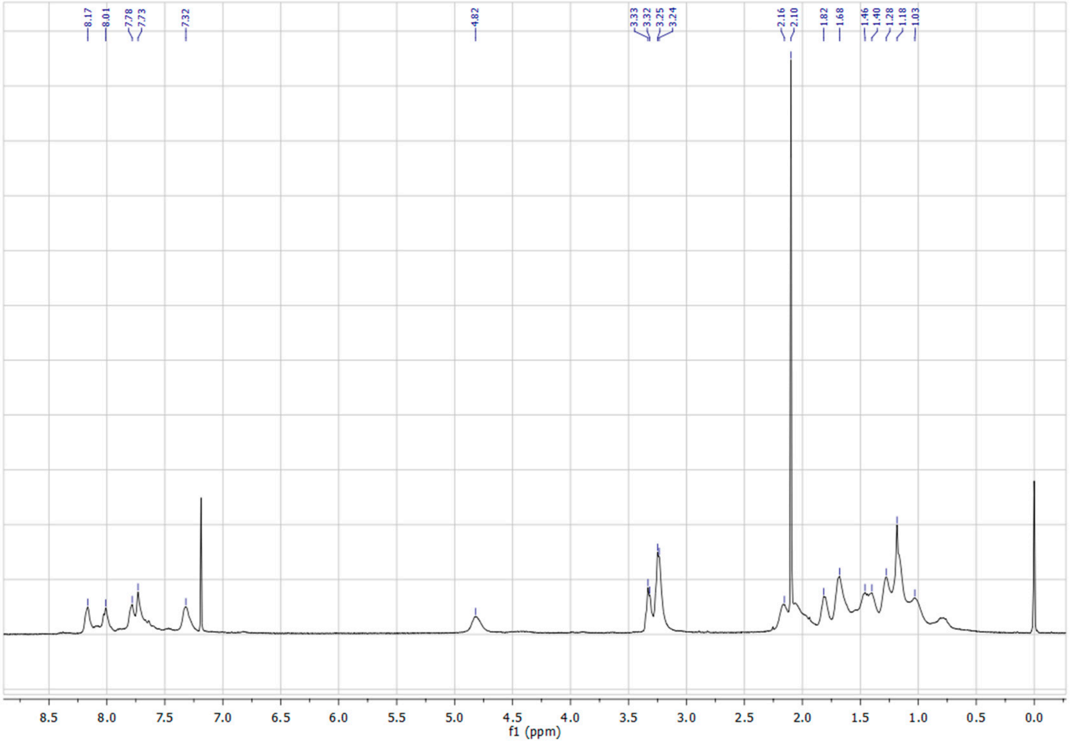

Figure S1. ^{1}H NMR spectra of CP entry in $CDCl_3$.

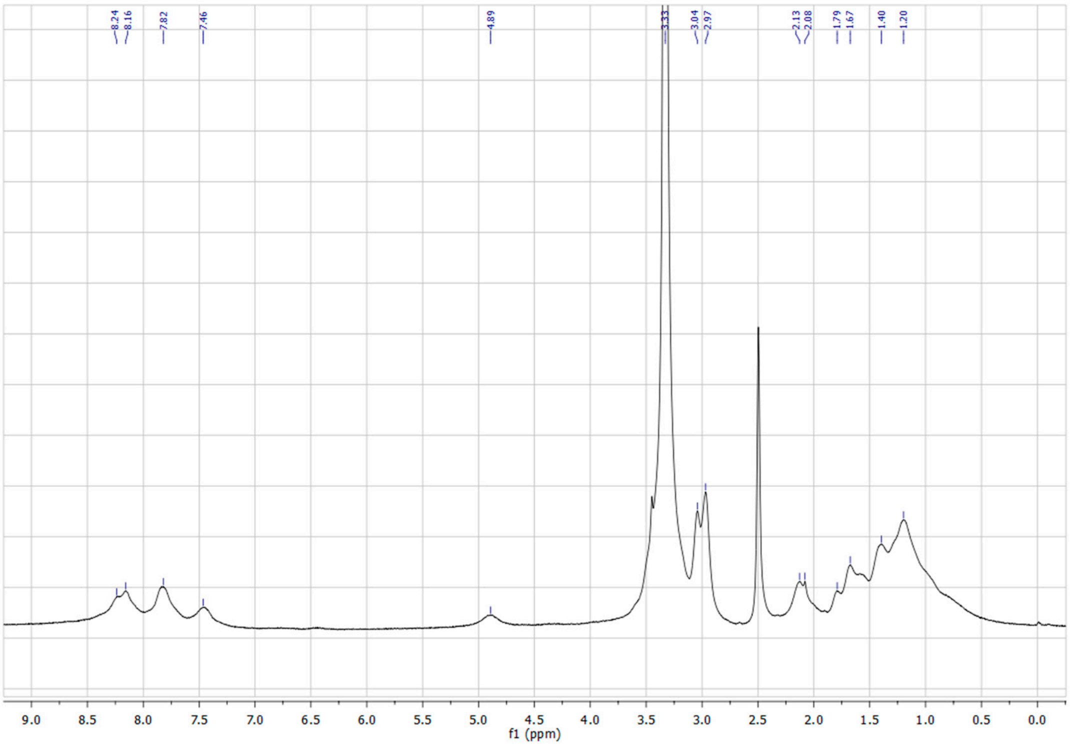

Figure S2. ^{1}H NMR spectra of CPE entry in DMSO-d_6.

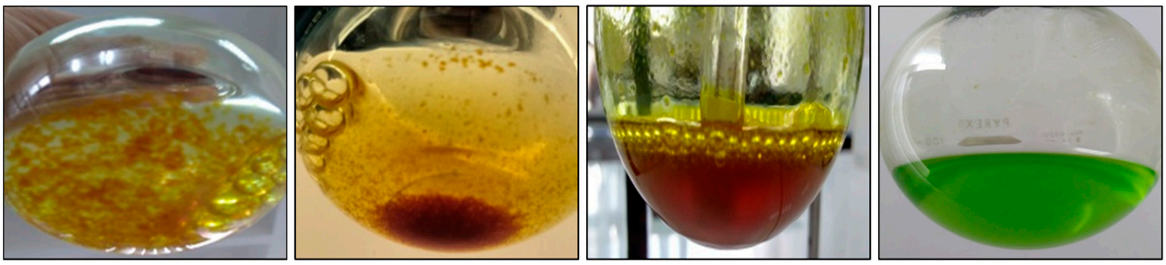

a) b) c) d)

Figure S3. Images of the reaction flasks containing the CuNPs and different amounts of CPE: a) 5 mg, b) 15 mg, c) 30 mg. d) Suspension b) after 48 h uncapped.

(a)

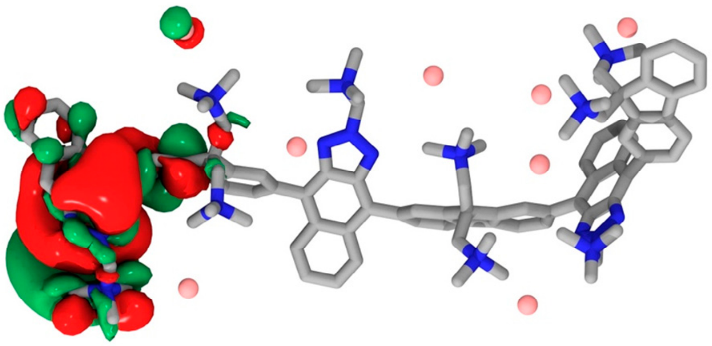

HOMO

(b)

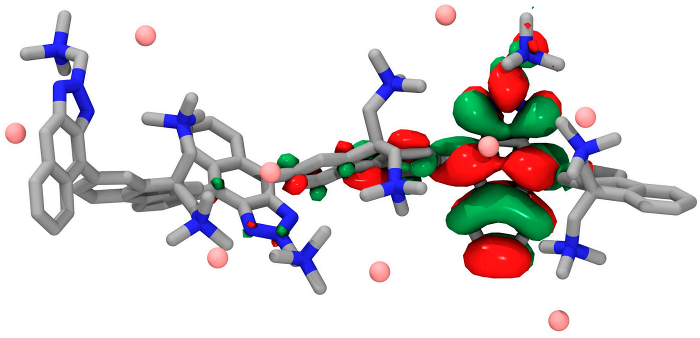

LUMO

Figure S4. (a) HOMO and (b) LUMO frontier orbitals of CPE at B3LYP/TZVP level.

(a)

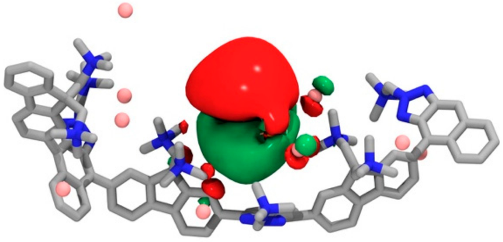

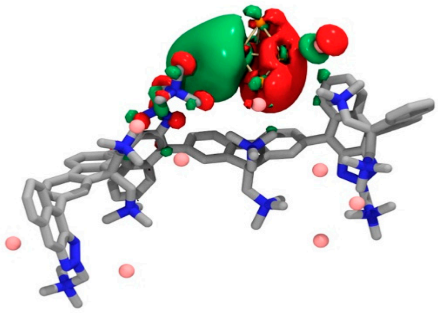

HOMO T-Cu_8-4 **HOMO T-Cu_8-5**

(b)

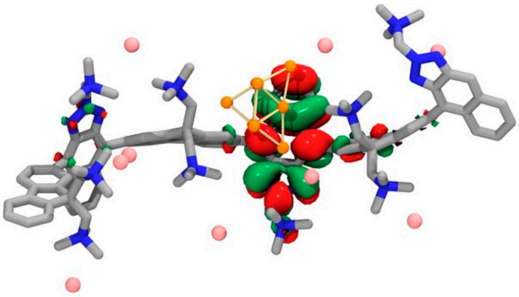

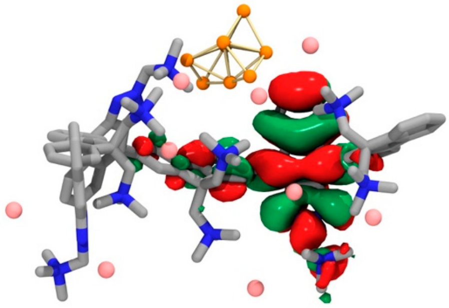

LUMO T-Cu_8-4 **LUMO T-Cu_8-5**

Figure S5. (a) HOMO and (b) LUMO frontier orbitals of complexes T-Cu_8-4 and T-Cu_8-5.

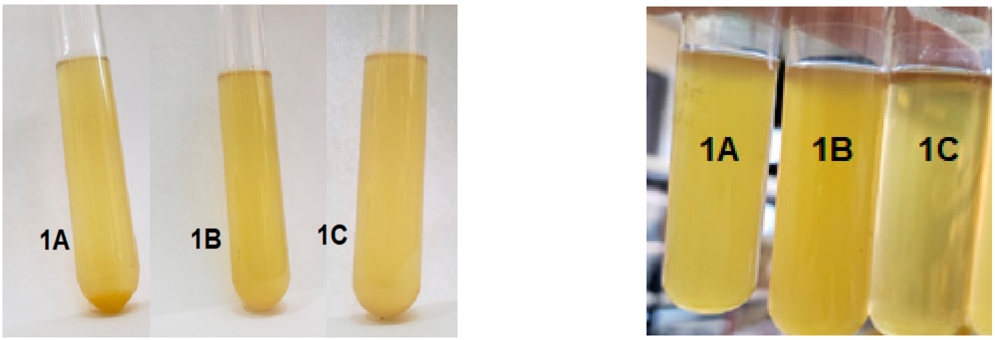

(a) **(b)**

Figure S6. Images of the *S. enteriditis* bacteria cultures incubated with CPE composite for 24 h (a) in the dark and (b) under irradiation.

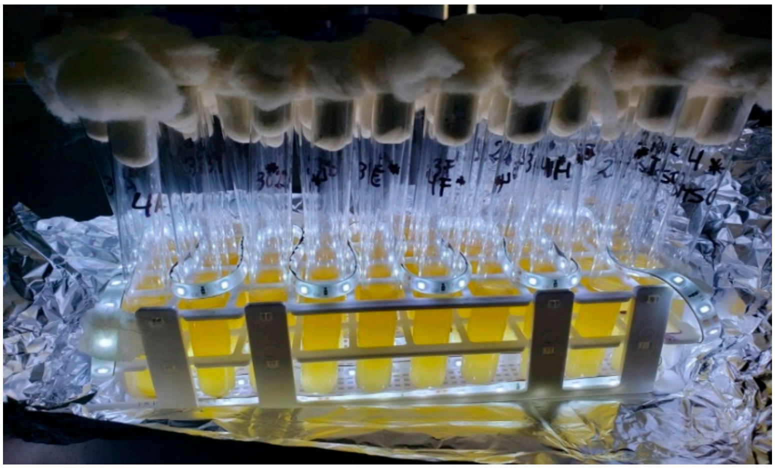

Figure S7. Image of an experiment run under white light irradiation.

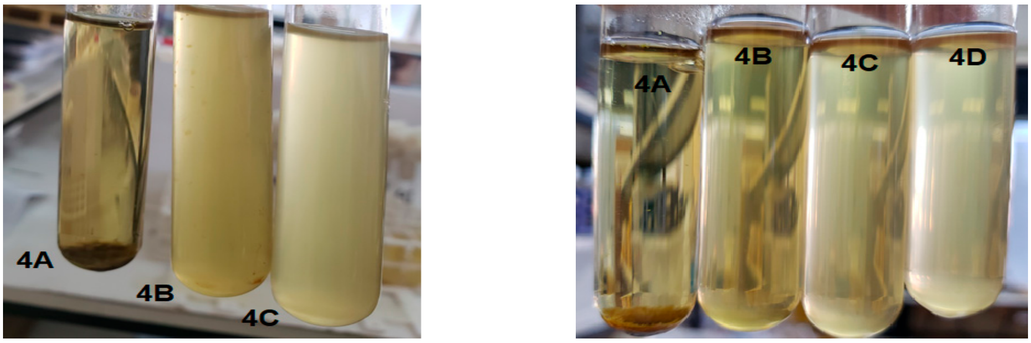

(a) **(b)**

Figure S8. Images of the *S. aureous* bacteria cultures incubated with CuNPs/CPE composite for 24 h (a) in the dark and (b) under irradiation.
